# Comparative bone healing with induced membrane technique (IMT) versus empty defects in septic and aseptic conditions in a novel rabbit humerus model

**DOI:** 10.1186/s12891-023-07031-3

**Published:** 2023-11-14

**Authors:** Claudia Siverino, Niels Vanvelk, Dirk Nehrbass, Dominic Mischler, Robert Geoff Richards, Mario Morgenstern, Stephan Zeiter, Daniel Arens, Thomas Fintan Moriarty

**Affiliations:** 1 AO Research Institute Davos, Clavadelerstrasse 1, Davos-Platz, 7270 Switzerland; 2 Center for Musculoskeletal Infections, Department of Orthopaedic and Trauma Surgery, University Hospital Basel, Basel, Switzerland

**Keywords:** Bone formation, IMT, Infection, Rabbit humerus defect model

## Abstract

**Background:**

Long bone defects resulting from primary trauma or secondary to debridement of fracture-related infection (FRI) remain a major clinical challenge. One approach often used is the induced membrane technique (IMT). The effectiveness of the IMT in infected versus non-infected settings remains to be definitively established. In this study we present a new rabbit humerus model and compare the IMT approach between animals with prior infection and non-infected equivalents.

**Methods:**

A 5 mm defect was created in the humerus of New Zealand White rabbits (*n* = 53) and fixed with a 2.5 mm stainless steel plate. In the non-infected groups, the defect was either left empty (*n* = 6) or treated using the IMT procedure (PMMA spacer for 3 weeks, *n* = 6). Additionally, both approaches were applied in animals that were inoculated with *Staphylococcus aureus* 4 weeks prior to defect creation (*n* = 5 and *n* = 6, respectively). At the first and second revision surgeries, infected and necrotic tissues were debrided and processed for bacteriological quantification. In the IMT groups, the PMMA spacer was removed 3 weeks post implantation and replaced with a beta-tricalcium phosphate scaffold and bone healing observed for a further 10 weeks. Infected groups also received systemic antibiotic therapy. The differences in bone healing between the groups were evaluated radiographically using a modification of the radiographic union score for tibial fractures (RUST) and by semiquantitative histopathology on Giemsa-Eosin-stained sections.

**Results:**

The presence of *S. aureus* infection at revision surgery was required for inclusion to the second stage. At the second revision surgery all collected samples were culture negative confirming successful treatment. In the empty defect group, bone healing was increased in the previously infected animals compared with non-infected controls as revealed by radiography with significantly higher RUST values at 6 weeks (*p* = 0.0281) and at the end of the study (*p* = 0.0411) and by histopathology with increased cortical bridging (80% and 100% in cis and trans cortical bridging in infected animals compared to 17% and 67% in the non-infected animals). With the IMT approach, both infected and non-infected animals had positive healing assessments.

**Conclusion:**

We successfully developed an in vivo model of bone defect healing with IMT with and without infection. Bone defects can heal after an infection with even better outcomes compared to the non-infected setting, although in both cases, the IMT achieved better healing.

**Supplementary Information:**

The online version contains supplementary material available at 10.1186/s12891-023-07031-3.

## Introduction

Regenerating bone and restoring limb function is a major clinical challenge in patients with long bone defects [1]. These defects may arise from severely comminuted injuries requiring removal of bone fragments, or from radical debridement of necrotic, fibrotic, and inflamed tissues due to fracture-related infection (FRI) [2, 3]. Although the surgical approach to repair large bone defects may be similar in both infected and non-infected cases, the underlying biological processes may be substantially different.

Numerous treatment options have been proposed for the reconstruction of bone defects. Options include non-structural bone grafting, vascularized fibular grafts, diaphyseal replacement, allograft, bone transport described by Ilizarov, and the induced membrane technique (IMT) introduced by Masquelet [4–6]. The IMT has gained popularity in the recent decades since the procedure relies on relatively standard surgical techniques, requires relatively less patient compliance and less frequent clinical follow up compared with other approaches [7].Nevertheless, several aspects of this technique are poorly understood, with some studies reporting poor outcomes in case of infected large bone defects and others failing to show benefits over the other clinically used techniques [8, 9].

The IMT is a 2-stage procedure, with the first stage consisting of implanting a polymethylmethacrylate (PMMA) spacer into the defect site, around which a local foreign-body response cascade leads to formation of an autologous induced membrane [10]. This stage can range from 4 to 96 weeks, depending on patient-specific circumstances [11]. Additionally, in case of infection or suspected infection, the PMMA is often loaded with antibiotics [12]. The second stage involves the lifting of the membrane to allow for spacer removal, followed by grafting autologous bone into the defect [13]. Healing usually continues for 8 to 12 months, with a mean defect gap of 4.4 cm [14]. The success rate in terms of union of the graft and resolution of the defect has been reported to be approximately 90% for adults [11] and 60% for paediatric patients [15]. However, postprocedural or recurrent infections have been reported in approximately 25% of patients receiving IMT, including surgical wound infections, and reactivation of initial infections [16]. The clinical literature about union rates in infected and non-infected defects is limited and is often based on low patient numbers [17–19]. Hence, clinical literature cannot clarify if the underlying biology of the origin of the defect (i.e., infected versus non-infected aetiologies) impacts on outcomes.

Several animal models have been developed to characterize the IMT and test the effect of various parameters on membrane formation and subsequent bone healing [20]. However, most of the described models did not involve infection as part of the experimental design.

In order to further study the influence of infection on bone regeneration with the IMT approach, a preclinical in vivo model would be a valuable resource to explore treatment concepts of bone defects caused by infection and acute trauma and facilitate studying the many clinically driven questions behind current treatment concepts. Therefore, the aim of this study is to develop a rabbit humerus model for the treatment of FRI including IMT and bone grafting. Bone healing is tested in the infected setting and compared to acute injury. Additionally, with this model it is possible to compare bone healing outcome between the IMT approach and the empty defect in both non-infected and infected scenarios.

## Materials and methods

The study was approved by the ethical commission of the canton Grisons in Switzerland (approvals 5_2019 and 4E_2020).

### Animals and experimental design

Healthy, skeletally mature, female New Zealand White rabbits of median age 20 months (range, 20–23 months) and median weight 3 kg (range, 3.0–5.5 kg), were used in this study. The animals were obtained from Charles River (Sulzfeld, Germany). In total, 53 animals were used in this study. Twenty-six animals had to be excluded. The first eight excluded animals were in the pilot and were operated using a 2.0 mm plate. Due to bending of the plate in the early postoperative days, the animals were excluded from the study and a 2.4 mm plate was used for the main study. Four were euthanized intraoperatively due to bone fracture; eight were excluded because they were culture negative at the first revision; three were excluded due to elbow joint infection, and finally three were excluded due to unexplained premature death not related to sepsis.

**Fig. 1:**
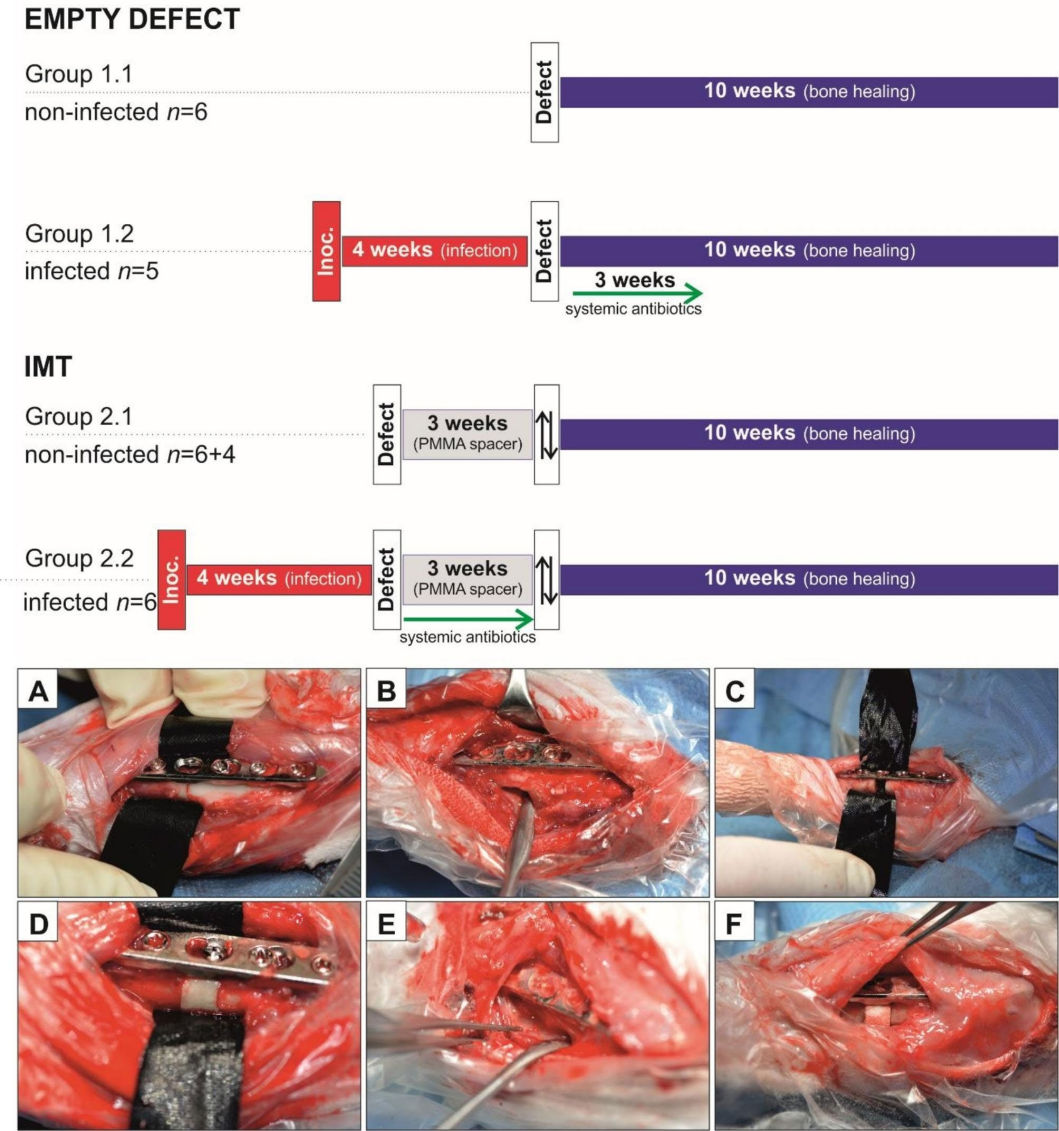
Schematic representation of the timeline and procedures involved in the empty defect and IMT approach. In the timeline (upper panel) the different stages of the surgery of the two groups, empty defect (Group 1) and IMT (Group 2) are represented. In Group 2.1, four additional animals (*n* = 6 + 4) were added to evaluate early membrane development at 3 weeks. The timeline of the study defines t = 0 as the time point when surgical interventions cease, and 10 weeks bone healing (blue box) starts. The box with the up and down arrow heads indicates the revision surgery where the polymethylmethacrylate (PMMA) spacer is removed and the bone substitute based on β-tricalcium phosphate is inserted. Systemic antibiotics (green arrow) = rifampicin 2 × 40 mg/kg and levofloxacin 2 × 30 mg/kg. The images below (AF) show the different stages of the surgery in the IMT group in case of infection. **(A)** First, the osteotomy is created, and the bone is infected with *S. aureus*. **(B)** After 4 weeks the wound is opened, visibly infected and necrotic tissue is removed, and **(C)** a 5 mm defect is created. **(D)** The defect is filled with a 5 mm PMMA spacer. **(E)** After 3 weeks an induced membrane has formed over the PMMA spacer. The spacer is removed, and **(F)** a bone graft substitute is inserted in the defect

The main study comprises 27 included animals divided in two main groups of rabbits with a bone defect that were treated either without any bone graft substitute, so-called “empty” group (Group 1, *n* = 11 in total), or those treated with the IMT and bone graft substitute (chronOS® Bone Graft Substitute, DePuySynthes) (Group 2, *n* = 16 in total) (Fig. 1). Both IMT and empty groups included rabbits that were either sterile, or intentionally inoculated with *S. aureus (*Group 1.1, *n* = 6 non-infected and Group 1.2, *n* = 5 infected; Group 2.1, *n* = 6 non-infected and Group 2.2 *n* = 6 infected). Four rabbits receiving the IMT were used for histological evaluation of the membrane around the PMMA spacer at an early stage, 3 weeks postoperatively (not inoculated) (Group 2.1, *n* = + 4).

Sample size calculation was performed before performing the study. The power calculation was done considering a difference in bone healing assessed by RUST score between the infected animals in the empty and IMT group of 20% with 80% power and *p* = 0.05. A *n* = 6 per group was calculated per group.

Blood was collected for white blood cell (WBC) analyses in the non-infected cohort: pre-OP, at revision (if applicable) and at the end of the study, while in the infected cohort WBC was analysed pre-OP, after 3 days, at 7 days from first surgery and every week until the end of the study.

During the study, numerous veterinarians, researchers, and technical stuff were involved. All the people involved in animal handling, surgery and bacteriological tests were blinded throughout the study. During outcome assessment all the researchers were also blinded. The main author was unblinded at completion of data collection in order to complete data analysis.

### Anaesthesia and surgical procedure

The rabbits were sedated and anesthetized according to the same protocols described in Arens et al. 2015 [21]. A skin incision was performed craniolaterally on the right humerus, exposing the lateral bone surface of the humerus. A 7-hole locking plate (Synthes; LCP Plate, straight, L 56 mm, stainless steel) was placed on the lateral surface of the humerus from the lateral epicondyle to the proximal deltoid ridge. The plate was maintained in place on the bone using a plate holding forceps with a swivel foot (Synthes; for plates 1.3 to 2.4 mm, speed lock). A drill guide (1.8 mm widened to 2.0 mm) was screwed into the most proximal plate hole to allow plate manipulation and placement of the implant. A 2.4 mm locking screw cut to a 0.3 mm length was placed in the 4th hole (central locking hole) to allow to pass the Gigly saw between the bone and the plate. The most distal screw was placed first; a bicortical hole was drilled with a 2.0 mm drill bit, the screw length measured, and the correct length locking screw (Synthes; ø2.4 mm, self-tapping with stardrive, various lengths) was inserted. The most proximal screw was then placed in the same fashion. The plate holding forceps was removed. The firstly placed distal and proximal screws were then tightened and the remaining 4 locking screws inserted.

#### Initial surgeries to assess plate thickness

In the initial part of the study a 7-hole 2.0 mm stainless steel locking plate (Depuy Synthes, Zuchwil, Switzerland) was used to fix the 5 mm empty defect (*n* = 8). Finite element (FE) analyses were performed to investigate the bending failure mode. Preoperative high-resolution peripheral quantitative computed tomography (HR-pQCT, XtremeCT, Scanco Medical AG, Brüttisellen, Switzerland) images of one animal were used to segment the bone shape and a post-operative scan was used to virtually position the plate by co-registering a CAD file of the plate to the scan. The models were meshed using linear tetrahedral elements using Simpleware M-2017.06 (Simpleware Ltd., Exeter, UK). To assess the influence of the plate design under axial loading, the 2 mm stainless steel plate and screws were compared with a 2.4 mm stainless steel plate and 2.4 mm screws. The screws were simplified as cylinders and bonded to the bone and plate interfaces. The bone was homogenously modelled with an elastic modulus of 20 GPa and the plate and screws with an elastic modulus of 180 GPa with a perfect-plastic behaviour at 690 MPa. The bone was constrained on the fossa radialis with only rotation allowed along the axis formed by the two condyles. An axial load on the humerus head centre was applied along the mechanical load axis from the head centre to the distal sulcus until the first occurrence of plasticity. The same load was then applied to the 2.4 mm plate and the axial displacement was compared to the 2.0 mm plate.

#### Empty defect

To create the defect, the central locking screw of 0.3 mm length was removed, and an ostectomy jig was fixed to the plate through placement of a central locking screw ensuring a standardized defect size of 5 mm. A protective Teflon coated foil was placed around the mid-diaphyseal humerus in the area of the intended ostectomy. A complete 5 mm ostectomy was created using two 0.45 mm Gigly saw wires (RISystem) placed within the previously installed guiding jig. The defect was left empty.

#### IMT approach

After creating the 5 mm defect, a 5 mm PMMA spacer (PALACOS® R bone cements, Heraeus) was inserted into the defect and held in place by insertion of a 1.5 mm self-tapping cortex screw (6 mm long, Synthes) (“step 1” of IMT approach). Three weeks after “step 2” surgery of the IMT approach was performed (Fig. 1: box with the up and down arrow heads). The membrane covering the PMMA spacer was incised, and subsequently gently elevated from the PMMA spacer which was removed. The defect was filled with bone graft substitute material consisting of pure β-tricalcium phosphate (chronOS® Bone Graft Substitute, DePuySynthes). In case of infected animals, gentamycin-loaded PMMA (PALACOS® R + G* bone cements, Heraeus, prepared according to manufacturer instructions) was used.

### Establishment of the infection

Anaesthesia and surgical procedure were performed as described above. In the infected animals, an osteotomy was created using two 0.45 mm Gigly saw wires (RIS.590.110) placed within the installed guiding jig. *S. aureus* (JAR 060131) was used to establish the infection. This strain is a clinical (rifampicin-susceptible) isolate from an infected human hip prosthesis and has previously been used in rabbit models of FRI [21]. *S. aureus* was recovered from frozen stock (-80 °C in 20% v/v glycerol) and cultured in tryptic soy broth (TSB, Oxoid, Basel, Switzerland) overnight at 37 °C with agitation at 100 rpm. On the day of surgery, the overnight culture was diluted to 1:20 in TSB and cultured as described before for 2 h. The bacterial culture was then adjusted to an optical density (OD) of 1 at 600 nm using a spectrophotometer (Multiskan, Thermo Fisher Scientific, Switzerland), corresponding to ~ 9.5 × 10^8^ CFU/ml. A total of 3 µl of the OD_600_ = 1 was pipetted over a collagen sponge (TissueFleece, Baxter) with a 3 mm diameter. The average CFU/sponge was ~ 2.8 × 10^6^ CFU. The collagen sponge was inserted at the osteotomy site. The OD_600_ = 1 was further diluted to OD_600_ = 0.25 using PBS and 6 µl of this suspension was pipetted over the two screw holes closest to the osteotomy site (~ 1.5 × 10^6^ CFU). The average of the total inoculum (collagen sponge and suspension) was 4.2 × 10^6^ CFU/animal (*n* = 11). The inoculum number was chosen according to previous studies in rabbits [22, 23].

### Surgical treatment: debridement of infected area

Four weeks after inoculation, the revision surgery (Stage 1) was performed. Visibly infected or necrotic tissue, abscesses, and pus were removed with blunt and sharp debridement and sampled for bacterial culture. All surfaces were then irrigated with 100 ml of sterile saline from a bulb syringe. The irrigation fluid was collected in single-use suction bags and sampled for bacterial culture. The osteotomy was widened to a 5 mm defect as described above and the removed bone was also collected for bacteriology. Rabbits from the infected sub-groups (1.2 and 2.2) were first infected and then randomly assigned to the empty defect or IMT group.

Animals from the IMT group infected (Group 2.2) underwent a second revision surgery (Stage 2) were the PMMA was removed and substitute with the bone graft as described above. During revision Stage 2, debridement tissue, PMMA spacer and irrigation fluid (100 ml) were collected and processed for bacteriology.

### Bacteriology of collected samples

At revision, the soft tissue, the debridement tissue, and the bone from the performed 5 mm defect were collected. Samples were weighed and then homogenized using in 10 ml phosphate buffer saline (PBS). The irrigation fluid (100 ml) and the other homogenized samples were sonicated for 3 min using Bandelin Ultrasonic water bath (Model RK 510 H). Bacteria were diluted to spot inoculate 10 µl of each dilution on blood agar (BA) plates (Oxoid) and incubated overnight at 37 °C to quantify the surviving population. Animals were considered as infected when at least one sample (soft tissue, debridement tissue, irrigation fluid or ostectomy) was culture positive. Identification of *S. aureus* in culture-positive samples was performed for at least one colony from each culture positive animal, using Staphaurex™ Latex Agglutination Test (Thermo Fisher) following comparison with the original *S. aureus* (JAR 060131).

### Systemic antibiotic treatment

The animals in the infected groups received oral systemic antibiotics twice a day 40 mg/kg Rifampicin (Labatec capsules, Labatec Pharma SA) and 30 mg/kg Levofloxacin (Sandoz Pharmaceuticals AG). Each day the animals received 1 g probiotics (Bene-Bac® Plus Pet Gel, Pet-Ag, Inc.) to minimize the gastrointestinal side effects of the antibiotics.

### Animal welfare

In the first five days postoperatively, the condition was recorded twice daily using a score sheet. Then daily until 7 days postoperatively. After that, a detailed assessment was made twice a week using the score sheet. If the score indicated that the welfare of the animals was impaired, the frequency of the score sheet assessment was adjusted accordingly. In addition to these score sheet assessments, the condition of the animals was checked by a veterinarian or an animal caretaker at least once a day.

### Radiographical examination

Radiographs of the right front leg were taken of all rabbits after the initial surgery, before and after revision surgeries, every week until the end of the study and after euthanasia. Contact radiograph was performed on the explanted humerus before processing for histology (cabinet x-ray systems, Faxitron, USA). Bone healing on radiographs was analysed using a modification of the radiographic union score for tibial fractures (RUST) originally developed by Whelan et al. [24]. This radiological scoring system was thereafter validated for the humerus. Each cortex on the anteroposterior and lateral radiograph is scored as 1 = no callus, 2 = callus present, fracture line visible, 3 = bridging callus, fracture line not visible. For animals treated with the IMT, a score of 3 was assigned in the presence of callus and absence of a radiolucent line between the bone and graft. All scores are summed up, resulting in a sum score ranging from 4 to 12. A RUST score ≥ 11 is considered as healed, a score < 9 is considered as not healed, a score 9–10 is considered neither union nor definite non-union [25]. Radiographs at 6 weeks and 10 weeks (considering t = 0 as starting point of bone healing) were used for RUST. The score was performed blinded, by one of the authors (N.V.).

### Histological processing and histopathological analyses

At the end of the study the rabbits were sacrificed, and the operated humerus was harvested and fixed in 70% methanol. Samples were processed for resin-embedding (with plate) in methyl methacrylate (MMA). Blocks were sectioned and ground down to a thickness of approximately 120 μm, and slides were stained with both, Giemsa and Eosin (G&E), as well as Brown and Brenn (BB). Semiquantitative histopathological analyses was performed blinded by a certified veterinary pathologist with a yes and no score for the bone cortexes bridging.

### Statistical analyses

The software GraphPad Prism 9 was used for all statistical analyses. First data were tested for normal distribution using: Anderson-Darling test, D’Agostino and Pearson test, Shapiro-Wilk test, Kolmogorov-Smirnov test. If one group from a dataset did not pass the normal distribution on one of these 4 tests, then the dataset was considered as not normally distributed. Analyses were done for the weight loss and WBC comparison between the different groups using a mixed-effects analysis with Šídák’s multiple comparisons test. For RUST statistical analyses were performed using Kolmogorov-Smirnov test or Mann Whitney test, *p < 0.05.

## Results

### Pilot study for optimal plate fixation

**Fig. 2:**
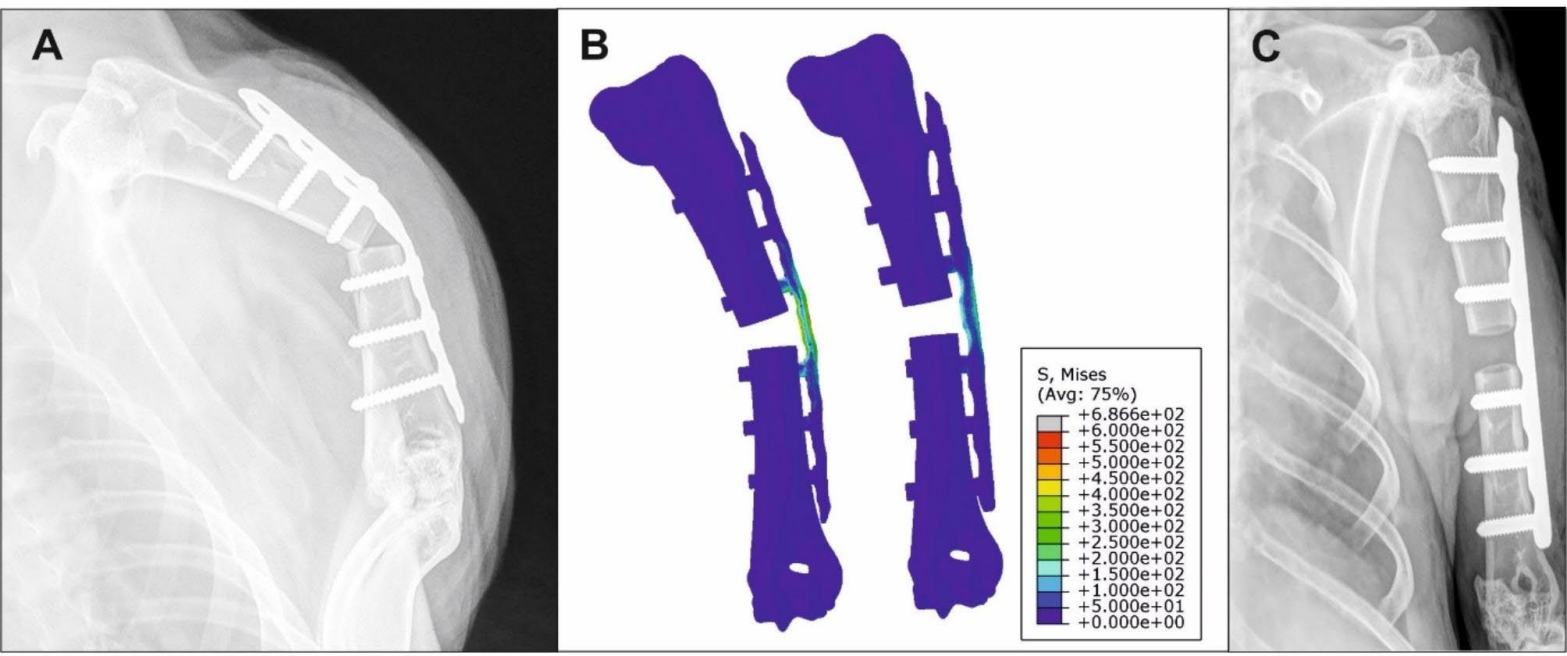
Radiographical examination and FE analysis of the 2.0 and 2.4 mm plate. **(A)** Radiograph of the 2.0 mm plate showing bending 1 week after surgery; **(B)** FE analysis of the 2.0 mm plate showed more pronounced bending (left) compared to the 2.4 mm plate (right). Note that for illustration purposes the FE deformations were scaled by a factor of 5; **(C)** Radiograph of the 2.4 mm plate 1 week after surgery

The 5 mm humeral defect was initially fixed with a 2.0 mm plate. In this first pilot batch of animals (*n* = 8), radiographical analysis one week postoperatively showed bending of the plate in two animals (Fig. 2, A). Therefore, a thicker 2.4 mm plate was investigated by finite element (FE) methods to determine if it could support the predicted loads in this model. Comparison of the two FE models showed more pronounced bending of the 2.0 mm plate with 231% higher axial displacement at 60 N compared to the 2.4 mm plate (Fig. 2, B). Furthermore, the 2.4 mm plate did not show any plasticity at this load level. Two animals were operated with the 2.4 mm plate and showed no signs of bending and good stabilization of the humerus defect (Fig. 2, C). These animals were included in the main study. The 2.4 mm plate was subsequently used for all further surgeries.

### Animal welfare

The rabbits experienced a maximal body weight loss of 10% throughout the entire study duration (Suppl. Figure 1, A). The infected animals in both empty defect and IMT groups experienced higher weight loss than the non-infected animals, although this was not present at all timepoints (*p* = 0.0352 at 4 weeks in empty defect group, and sporadically in the IMT groups). No animals had to be excluded or prematurely euthanized due to body weight loss exceeding abruption criteria (> 20%) or because of other conditions such as fever, loss of appetite or impaired general behaviour. White blood cell (WBC) analysis showed higher counts in the infected groups compared to the non-infected animals, but this did not reach statistical significance (Suppl. Figure 1, B).

### Evaluation of infection induction and resolution by quantitative bacterial culture

**Fig. 3:**
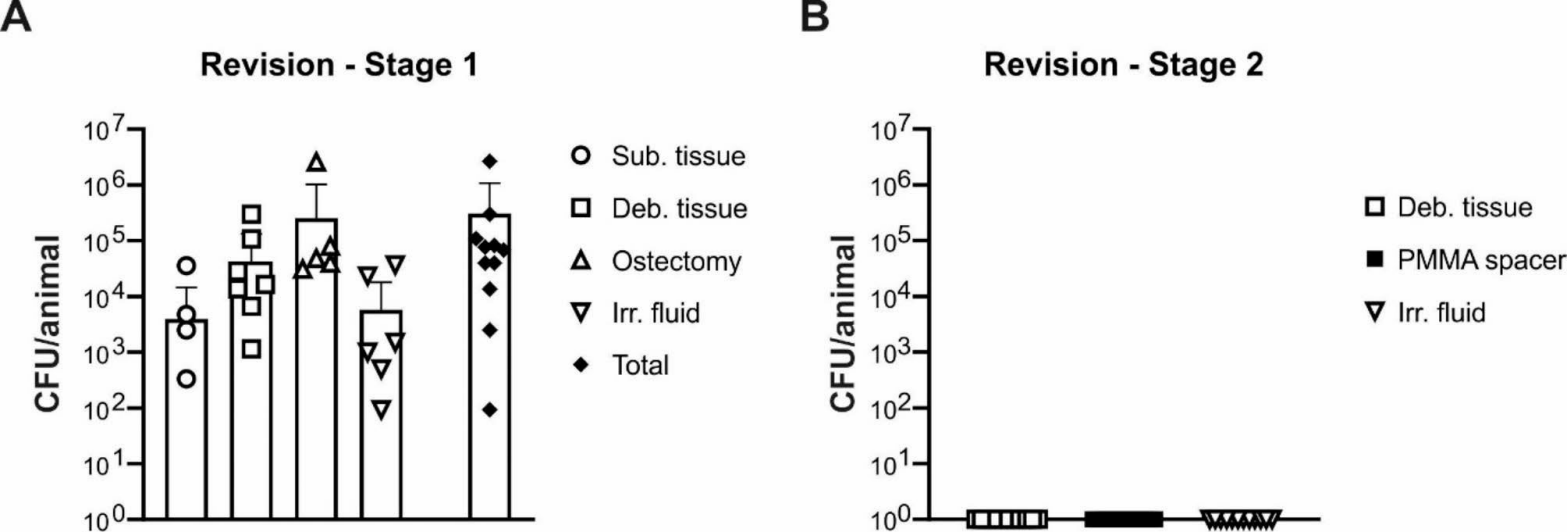
Bacteriology of retrieved samples during the revision surgery stage 1 and stage 2. **(A)** At 4 weeks post inoculation, rabbits (*n* = 11) underwent a first revision surgery, where the debridement tissues and the irrigation fluid used for lavage of the fracture area were collected and quantified. **(B)** At the second revision surgery (*n* = 6 from the IMT group, 2.2), debridement tissue, PMMA spacer and the irrigation fluid were collected and cultured. Culture-negative samples were arbitrarily assigned a value of 1 for the purposes of displaying on a log_10_ axis. Error bars indicate standard deviation (SD)

All the infected animals from the empty defect and IMT groups underwent a first revision surgery, in which debrided materials were submitted for bacterial culture. The samples collected during the first revision surgery (before any debridement or antibiotic therapy) confirmed the presence of bacteria in all animals (Fig. 3, A). Animals from the empty group did not go through the second revision. In all seven animals of the IMT group that were infected, tissue cultures showed complete clearance of the infection at the second revision (Fig. 3, B).

### Bone healing in the empty defect group

#### Radiographical examination

**Fig. 4:**
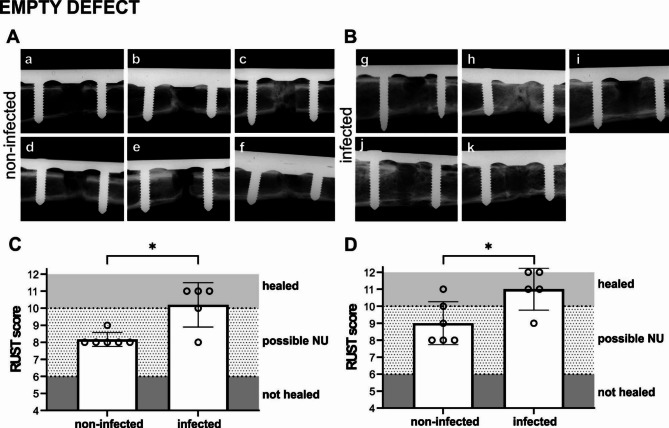
Radiographical images (anteroposterior) of the empty defect group (non-infected and infected) at the end of the study. **(A)** Empty defect non-infected, *n* = 6; **(B)** Empty defect infected, *n* = 5. **(C, D)** Quantification of bone healing in the two groups using RUST of the anteroposterior and lateral radiographs of the rabbit humerus at **(C)** 6 weeks and **(D)** end of the study. A RUST value > 10 is considered as healed osteotomy, RUST value < 6 is considered as not healed osteotomy, RUST value between 6–10 is considered neither union nor definite non-union. Statistical analyses were performed using Mann Whitney test, *p < 0.05). Error bars indicate SD

The direct comparison of bone healing in the non-infected vs. infected animals at 6 weeks and euthanasia in the empty defect group, is shown in Fig. 4. Radiographical imaging shows bone healing differences between animals in each group (Fig. 4 A, B). RUST values show that the infected animals overall have a significantly higher bone healing at 6 weeks (*p* = 0.0281) (Fig. 4, C) and at the end of the study (week 10) (*p* = 0.0411) (Fig. 4,D) compared to the non-infected animals.

#### Histopathological analyses

**Fig. 5:**
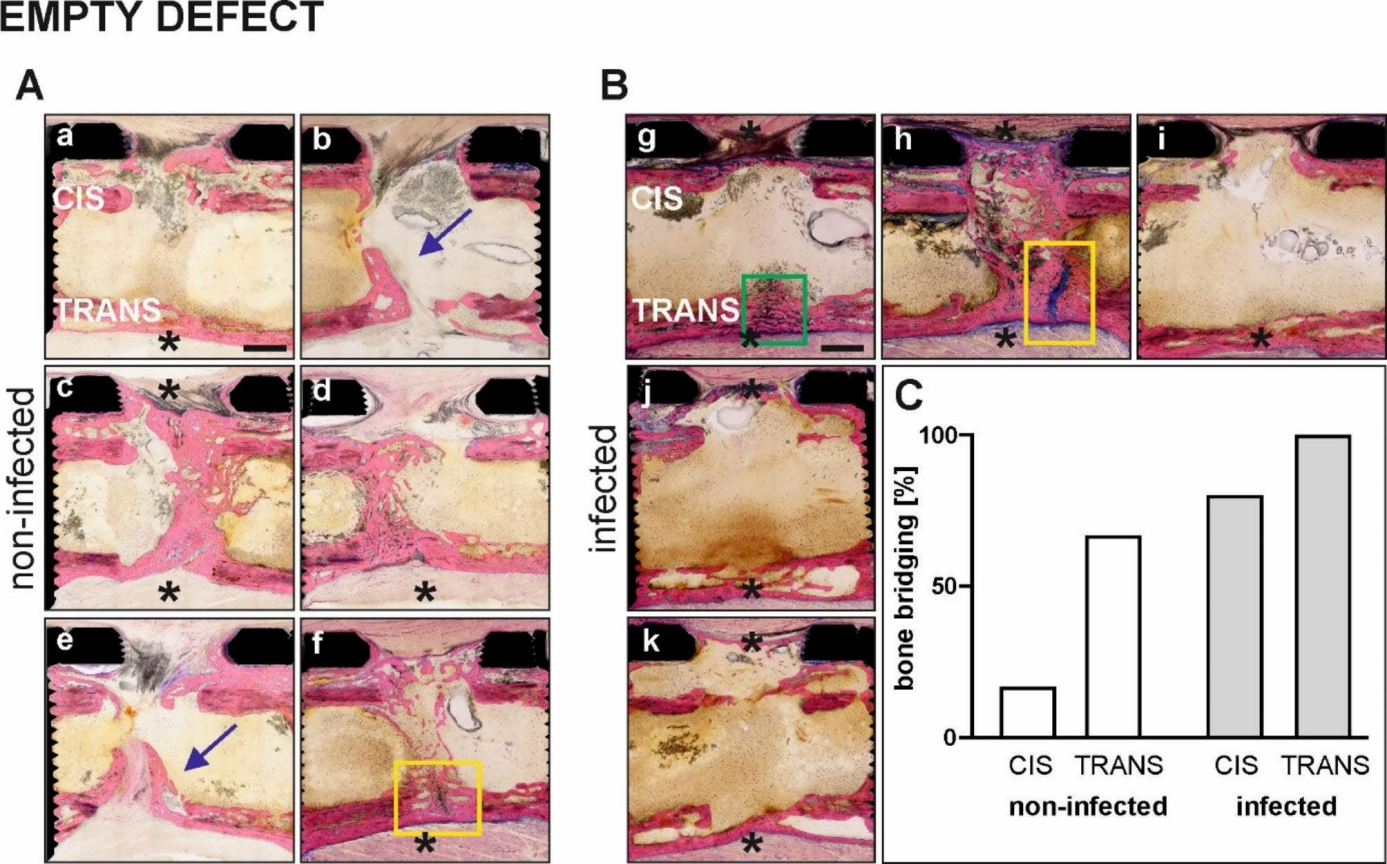
Overview microphotographs of the ostectomy site and graph showing the incidence of cortical bridging of the empty defect group (non-infected vs infected). **(A, a-b)** non-infected animals (*n* = 6); **(B, g-k)** infected animals (*n* = 5). Note the bony bridging via (CIS and/or TRANS) cortex in sections marked by asterisk. Blue arrows indicate new bone in the medullary area. Yellow and green boxes shown cartilaginous tissue. Graph showing the percentage of animals with CIS and TRANS cortical bridging in the non-infected and infected groups. Scale bar a-k 2 mm

Giemsa Eosin-stained sections and semiquantitative analysis of the defect sites in the empty group are shown in Fig. 5. Defects of the non-infected humeri show less cortical bridging, especially at the CIS side (1/6 CIS side; 4/6 TRANS side) while all the infected animals show complete cortical bridging either at the CIS or TRANS side (4/5 CIS side; 5/5 TRANS side). The non-infected animals show more new bone in the medullary area shown by the blue arrows (Fig. 5, b and e). The histopathological evaluation also reveals the presence of cartilaginous tissue in a few animals (yellow boxes in Fig. 5, f and h). This intense blue staining within the osteotomy gap at the TRANS cortex is interpreted as a result of instability and has to be differentiated from the purple-blue stained areas of ongoing endochondral ossification (green box in Fig. 5, g).

### The IMT approach

#### Evaluation of the masquelet membrane formed after 3 weeks over the PMMA spacer

**Fig. 6:**
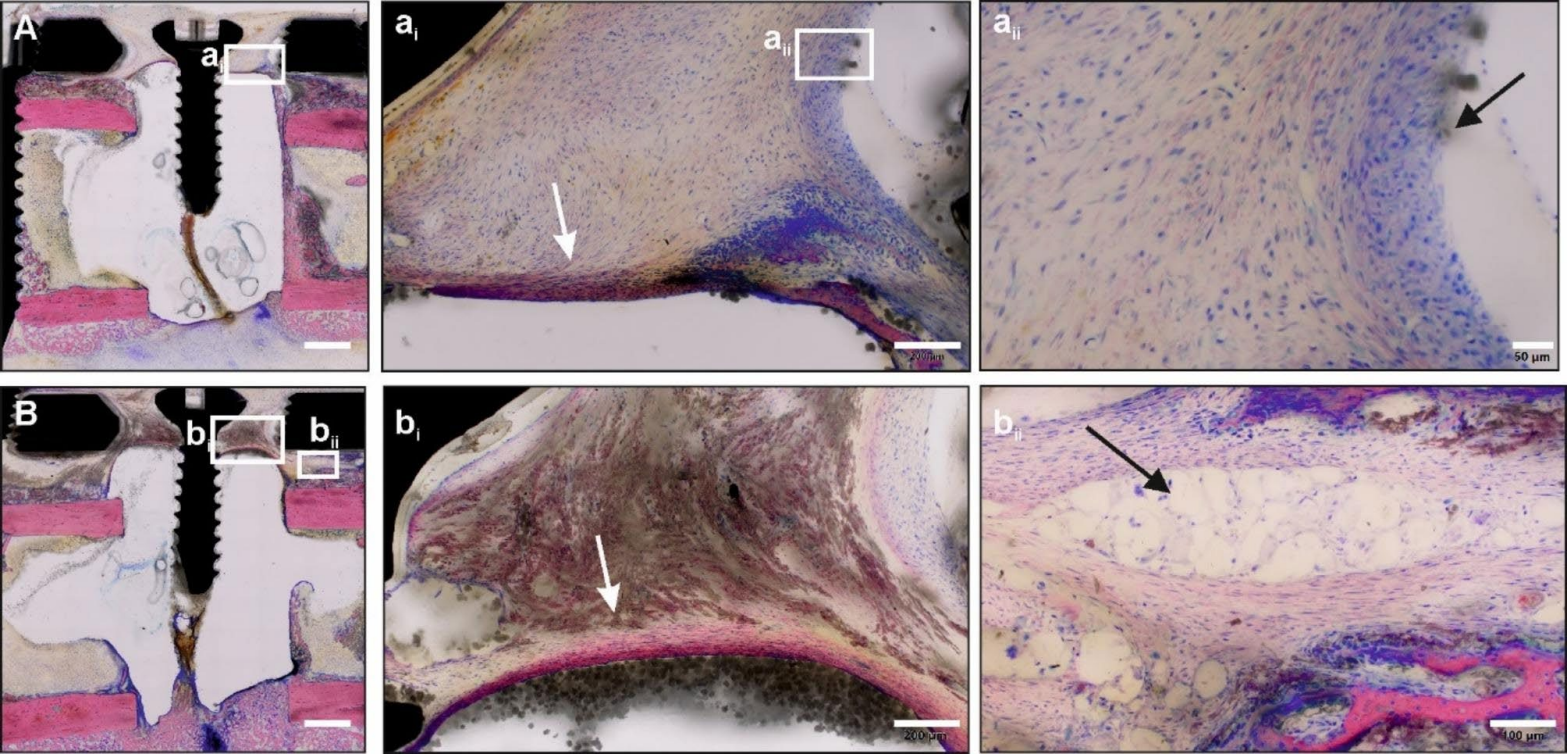
Representative microphotographs of the of the induced membrane formed at the interface with the PMMA spacer 3 weeks after IMT surgery. **(A, B)** 2 representative animals of the group 2.1 (*n* = 4, non-infected – membrane evaluation). A higher magnification of the marked insets in images A and B are given on the right side of the images (a_i_, a_ii_ and b_i_, b_ii_). PMMA-embedded, GE-stained, thick-sections, scale bar 2 mm (A, B); or 200 μm (a_i_, a_ii_), 100 μm (b_ii_) and 50 μm (a_ii_). White arrows mark fibrous tissue (a_i_, b_i_), black arrows indicate early state granulomatous inflammation (a_ii_, b_ii_)

The development of the membrane over the PMMA spacer at an early time point (3 weeks after initial surgery) was evaluated histopathologically in four non-infected animals of group 2.2 (Fig. 6). As reported by literature, a fully developed membrane forming over the PMMA spacer is composed of a fibrous capsule with both loose and/or dense fibrous tissue accompanied by granulomatous inflammation [7]. However, at 3 weeks the membrane is not fully developed in this model and consists at this early stage of loose and dense fibrous tissue (Fig. 6 a_i_ and b_i_) and nearly no acute inflammatory changes (e.g., hypervascularization, edema, granulocytic cell infiltration, necrosis) close to the membrane. The presence of PMMA debris close to the PMMA spacer resulted in early-stage formation of low-grade granulomatous inflammation (Fig. 6, a_ii_ and b_ii_).

#### Radiographical examination of bone healing in the IMT group

**Fig. 7:**
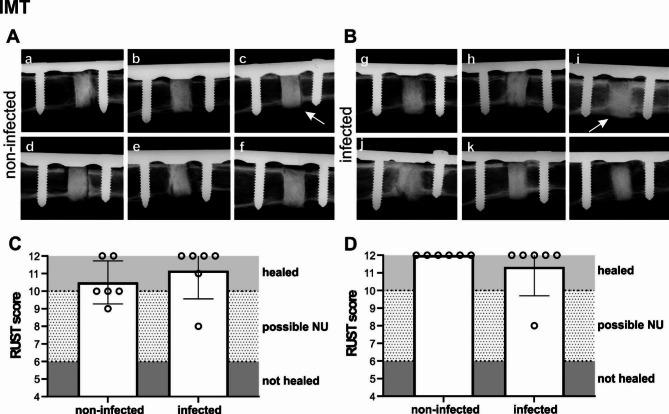
Radiographical images (anteroposterior) of the non-infected and infected IMT groups at the end of the study. **(A)** IMT non-infected, *n* = 6; **(B)** IMT infected, *n* = 6. **(C, D)** Quantification of bone healing in the different groups using RUST of the anteroposterior and lateral radiographs of the rabbit humerus at **(C)** 6 weeks and **(D)** end of the study. A RUST value > 10 is considered as healed osteotomy, RUST value < 6 is considered as not healed osteotomy, RUST values between 6–10 are considered neither union nor definite non-union. White arrows indicate representative cases of bone forming around the outside of the scaffold. Statistical analyses were performed using Mann Whitney test, no significant differences were measured. Error bars indicate SD

The overview of the radiographs of the non-infected and infected animals of the IMT group at the end of the study is shown in Fig. 7. In all the animals, independent of the group, the defect area filled with the bone substitute for 10 weeks showed new bone forming around the outside of the scaffold (Fig, 7 A and B, white arrow representative cases). Due to the presence of bone graft substitute, the difference in bone healing between the groups is not easily discernible by contact radiograph (Fig. 7, A and B). Bone healing in the non-infected and infected group treated with IMT were nonetheless scored using RUST. The two groups show similar bone healing at 6 weeks from revision surgery and at the end of the study. Interestingly, only 2 of the 6 defects of the non-infected IMT group were scored as healed at 6 weeks compared to 5/6 in the infected group (Fig. 7, C). At the end of the study, both groups showed complete healing with mean RUST scores of 12 and 11.43 (non-infected and infected, respectively) (Fig. 7, D).

#### Evaluation of bone healing after IMT by histopathology

**Fig. 8:**
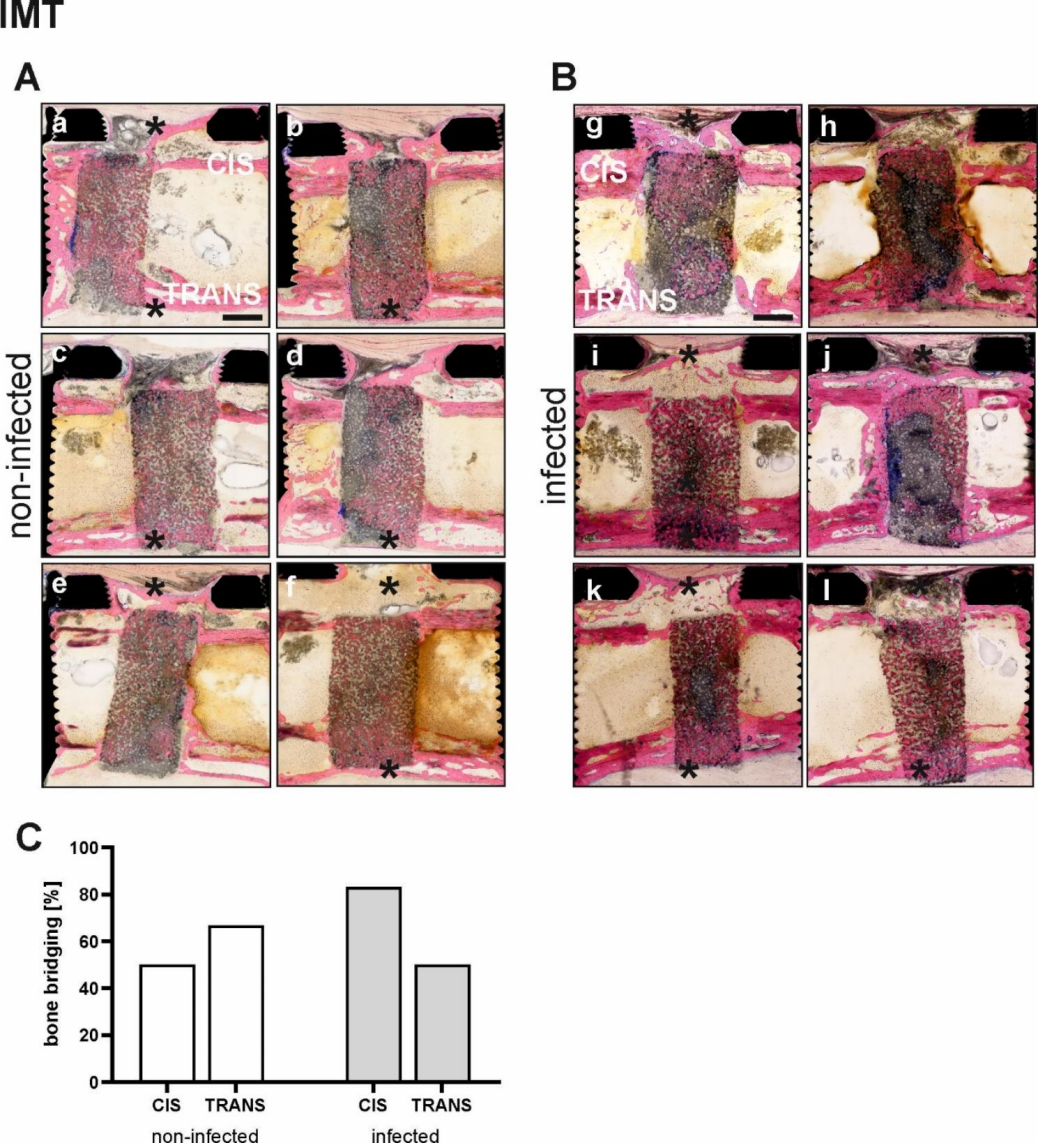
Overview microphotographs of the ostectomy site as well as graph showing the incidence of cortical bridging of the IMT defect group (non-infected vs. infected). **(A)** a-f non-infected animals (*n* = 6) and **(B)** g-l infected animals (*n* = 6). **(C)** Graph showing the percentage of animals with CIS and TRANS cortical bridging in the non-infected and infected groups

Overview images and healing assessments of the defect site of all animals treated with IMT are shown in Fig. 8. Cortical bridging was achieved more often in the infected animals (5/6, 83.3%) compared to the non-infected (3/6, 50%) at the CIS cortex. In contrast, the TRANS cortex had similar bridging in the infected and non-infected groups (3/6 infected and 4/6 non-infected, 50% and 66.7% respectively). Representative G&E images in Suppl. Figure 2A and Suppl. Figure 2B show the bone formed within the bone substitute (Suppl. Figure 2A, a_i_ and Suppl. Figure 2B, c_i_) and the membrane (Suppl. Figure 2A, a_ii_ and b_i_). At euthanasia, ten weeks after exchanging the PMMA spacer for the bone graft substitute, a fully developed Masquelet membrane was observed above the bone graft substitute, comprising of a fibrotic capsule with loose and dense fibrous tissue (blue arrows in Suppl. Figure 2A, a_ii_, b_i_), and also presence of multinucleated giant cells with grey cytoplasmic material as a sign of chronic granulomatous inflammation against PMMA (Suppl. Figure 2B, c_ii_ and d_i_, d_ii_).

Histological evaluation using BB staining of all infected humeri showed the presence of few, if any, bacteria in areas close to the defect in all animals of the empty defect group (5/6) and of the IMT group (5/5) (Suppl. Figure 3). However, this stain cannot differentiate live from dead bacteria.

## Discussion

The IMT is one of the most well-established methods for the reconstruction of long bone defects [26]. Compared to bone transport, the IMT is often preferable as it uses relatively standard surgical techniques and implants, limits the need for high patient compliance, and minimizes the frequency of clinical follow-up. Nonetheless, several clinical studies evaluating the effectiveness of the method have shown inconsistent results with either successful results [27] or with incidences of non-union or re-infection in both aseptic and septic settings [11, 28, 29]. Because of the large diversity within the patient populations receiving IMT, many aspects of the procedure, such as spacer material, use of local antibiotics, and time interval between revision surgeries, are not easily evaluated in human clinical trials. Animal studies offer the possibility to evaluate those factors contributing to the outcome of the IMT under controlled conditions. The aim of this study was to develop an IMT model in the rabbit humerus and compare outcomes in terms of bone healing in infected and non-infected settings. Overall, the model was successfully established, and comparative outcomes suggest that bone healing may be superior with the IMT approach compared to empty defects, and that infection may in fact not hinder the bone healing outcome but rather improve it.

Our findings in the empty defect cohort, i.e., in the absence of any spacer or bone void filler, suggest that healing progression was improved by the prior infection. Histopathology and radiography both revealed higher bone healing when comparing parameters such as new bone formation and bone bridging in the CIS and TRANS cortical areas in the infected animals compared to non-infected equivalents. Increased bone formation in the presence of bacteria has previously been described, with non-viable bacteria leading to more favourable bone formation with enhanced periosteal bone formation and increased cortical thickening in a rabbit tibia model [30]. Similarly, experimental studies in rabbits have shown that the presence of non-viable bacteria or *S. aureus* cell-wall-derived lipoteichoic acid (LTA) with a suboptimal dose of bone morphogenetic protein 2 (BMP2) can lead to ectopic bone formation suggesting a pro-osteogenic effect [30]. It has been proposed that the inflammatory response to bacteria leads to pro-inflammatory cytokines acting in synergy with osteoinductive signals thereby enhancing osteogenic differentiation [31]. Therefore, an inflammatory milieu distant from the infection site may stimulate osteogenesis, while in the vicinity of the bacterial burden the same inflammatory response may induces osteolysis or failure of fracture healing [32]. It may well be, therefore, that the infection initiated in our rabbit model led to a persistence of a mild inflammatory response that was localised to the defect area and induced greater bone formation within the defect site.

Clinically, large bone defects are rarely if ever left empty. The clinical approach often used in such contexts, the IMT approach, was next modelled in our study. One of the challenges in evaluating the outcomes in the IMT groups is that the biomaterial itself can obscure bone formation when observed radiologically. Histopathology could, however, show new bone ingrowth at the CIS and TRANS cortical side and in the medullary area of IMT-treated rabbits. In contrast to the rabbits in the empty defect group, there was no observable consistent increase in bone healing in the previously infected group compared to the non-infected group when treated with IMT. A higher grade of bone bridging was, nevertheless, observed in the CIS cortical side in the infected group compared to the non-infected group. Although the defect site was not fully regenerated with remodelled bone and fully resorbed bone substitute at the investigation time of 10 weeks, both radiological and histopathological scores showed that the IMT resulted in healed and bridged defects in both infected and non-infected settings with higher scores compared to the empty defect. The full assessment of bone healing in these defects would require mechanical testing, however, these data suggest improved outcomes in terms of cortical bridging after IMT in septic and aseptic conditions compared to the empty defect.

One of the central aspects of the IMT, which may be at least partially responsible for the increased healing rate in the IMT treated rabbits compared to those with empty defects (both infected and non-infected) is the membrane that is induced around the spacer. The induced membrane contains several pro-regenerative mediators which enhance bone healing [33]. In fact, detailed investigation of membrane compositions revealed presence of collagen type I, III and aggrecan but also, growth factors as BMP2, transforming growth factor beta (TGFβ), vascular endothelial growth factors (VEGF) and inflammatory and anti-inflammatory factors like interleukin (IL) 6, 8 and 10 and tumour necrosis factor alpha (TNFα) [34–36]. Additionally, cells isolated from the membrane of bone defect patients treated with IMT approach, showed multipotency with chondrogenic, adipogenic and osteogenic differentiation similar to periosteum derived cells [37]. In our study the membrane was not fully developed after 3 weeks since it consisted of an early-stage formation of loose and dense fibrous tissue with only a low-grade granulomatous inflammation close to the membrane. In contrast, by the end of the study at 10 weeks, the membrane was fully developed in both septic and aseptic conditions with a chronic granulomatous inflammation at the interface. Further investigations could focus on detailed molecular characterization of the membrane to evaluate how the infection induced-inflammatory environment influences membrane composition and potentially to identify materials or factors that may stimulate an accelerated membrane formation.

Several animal models have already been used to investigate different aspects of the IMT [20]. Few studies compared the IMT approach with an empty defect, in relation to bone healing outcomes [38–41]. Fewer models incorporate the second stage of the procedure, beyond the initial membrane characterization [35, 42, 43] and continued to a second stage revision using bone graft or bone substitute biomaterials assessing bone healing by radiography and micro-CT [44, 45]. Finally, most of the described models did not involve infection as part of the experimental design, even though infection is a major cause of bone defects, and the local tissue conditions secondary to infection may be substantially different from acute, non-infected defects. The few studies that did include infection followed by IMT were performed in a rat model with a femoral critical size defect fixed with a custom fixation device and inoculated with *S. aureus* [46, 47]. The results showed the clearance of the infection in most of the animals when using an antibiotic loaded cement spacer compared to animals treated with cement without antibiotics. However, the outcomes focused only on the in vivo bacterial recovery and histological and gene expression analyses of the growth factors or inflammatory cytokines contained in the membrane surrounding the defect, but not on bone healing itself and did not compare outcomes with empty defects [46, 47]. However, beyond what has already been published, our model replicates the clinical scenario closely to allow for better investigation of the different factors influencing the IMT outcomes. We included a 2-stage procedure including the implantation of a PMMA spacer and bone grafting using a beta-tricalcium phosphate-based bone substitute. In an advance from previous studies, we included infection with *S. aureus* and as fitting to conventional clinical education [2, 48], we also properly managed the infection with expedient debridement, adequate dead space management and systemic and local antibiotic treatment. Additionally, we compared the outcomes of the infected groups to the aseptic condition not only by radiography but also by histopathology. To conclude, the rabbit defect model highly replicates the clinical IMT approach and could be used to further investigate the effect of various factors such as different spacers, local antibiotic treatment, or new biomaterials on bone healing and/or the eradication of the infection.

This study has a number of limitations. First of all, the application of the IMT in the case of infection would not proceed without proven eradication of the infection. From the infection treatment perspective, we applied as clinical practice often recommends, PMMA as local antimicrobial delivery system and for the dead space management after debridement [12] and applied systemic antibiotic therapy immediately. This was followed by scheduled spacer removal, and placement of the bone graft substitute. All biopsies and debrided tissues were culture negative at this second revision, suggesting clearance of the infection, however, we cannot unequivocally claim infection eradication as there is a chance of false negatives, and the final outcomes did not include quantitative bacteriology. Nevertheless, histological analysis revealed few if any bacteria at euthanasia and the culture negative biopsies support proceeding to bone grafting. In addition, young people have a generally greater potential to clear the infection and a faster bone healing compared to elderly people [32, 49]. This poses a limitation in this study and to the extent to which the outcomes can be compared to the human situation. However, this study offers the chance to perform more controlled studies without confounding variables which can affect human studies. Another important limitation is the fact that the bone graft hinders proper evaluation of the bone healing since radiography cannot easily separate bone graft from new bone. Finally, the RUST score is not usually performed in case of plating osteosynthesis, however, we adapted the scores to provide an assessment of healing, although these results should be interpreted with caution.

## Conclusion

The novel animal model described in this study, replicating the IMT in both an infected and non-infected setting, includes several important features of the clinical setting. It represents an optimal pre-clinical model to study several aspects of the IMT in detail. Certainly, a better understanding of how the membrane’s characteristics are influenced by infection and how those factors affect healing outcomes remains crucial. In this study we could prove that bone defects can heal after an infection with even better outcomes compared to the non-infected setting, although in both cases, the IMT achieved better healing.

### Electronic supplementary material

Below is the link to the electronic supplementary material.

Supplementary Material 1

## Data Availability

The datasets generated during and analysed during the current study are not publicly available due to internal data storage restriction but are available from the corresponding author on reasonable request.

## References

[1] Puetzler J, Zalavras C, Moriarty TF, Verhofstad MHJ, Kates SL, Raschke MJ (2019). Clinical practice in prevention of fracture-related Infection: an international survey among 1197 orthopaedic trauma surgeons. Injury.

[2] Metsemakers WJ, Morgenstern M, Senneville E, Borens O, Govaert GAM, Onsea J (2020). General treatment principles for fracture-related Infection: recommendations from an international expert group. Arch Orthop Trauma Surg.

[3] Moriarty TF, Metsemakers WJ, Morgenstern M, Hofstee MI, Vallejo Diaz A, Cassat JE (2022). Fracture-related Infection. Nat Rev Dis Primers.

[4] Roddy E, DeBaun MR, Daoud-Gray A, Yang YP, Gardner MJ (2018). Treatment of critical-sized bone defects: clinical and tissue engineering perspectives. Eur J Orthop Surg Traumatol.

[5] Ilizarov GA, Lediaev VI (1969). [Replacement of defects of long tubular bones by means of one of their fragments]. Vestn Khir Im I I Grek.

[6] Masquelet AC, Fitoussi F, Begue T, Muller GP (2000). [Reconstruction of the long bones by the induced membrane and spongy autograft]. Ann Chir Plast Esthet.

[7] Alford AI, Nicolaou D, Hake M, McBride-Gagyi S (2021). Masquelet’s induced membrane technique: review of current concepts and future directions. J Orthop Res.

[8] Rohilla R, Sharma PK, Wadhwani J, Das J, Singh R, Beniwal D (2022). Prospective randomized comparison of bone transport versus Masquelet technique in infected gap nonunion of tibia. Arch Orthop Trauma Surg.

[9] Gupta GK, Majhee AK, Rani S, Shekhar S, Prasad P, Chauhan G (2022). A comparative study between bone transport technique using Ilizarov/LRS fixator and induced membrane (Masquelet) technique in management of bone defects in the long bones of lower limb. J Family Med Prim Care.

[10] Masquelet AC (2017). Induced membrane technique: pearls and pitfalls. J Orthop Trauma.

[11] Morelli I, Drago L, George DA, Gallazzi E, Scarponi S, Romano CL (2016). Masquelet technique: myth or reality? A systematic review and meta-analysis. Injury.

[12] Metsemakers WJ, Fragomen AT, Moriarty TF, Morgenstern M, Egol KA, Zalavras C (2020). Evidence-based recommendations for local antimicrobial strategies and Dead Space Management in fracture-related Infection. J Orthop Trauma.

[13] Mauffrey C, Hake ME, Chadayammuri V, Masquelet AC (2016). Reconstruction of Long Bone Infections using the Induced membrane technique: Tips and tricks. J Orthop Trauma.

[14] Raven TF, Moghaddam A, Ermisch C, Westhauser F, Heller R, Bruckner T, Schmidmaier G (2019). Use of Masquelet technique in treatment of septic and atrophic fracture nonunion. Injury.

[15] Auregan JC, Begue T, Rigoulot G, Glorion C, Pannier S (2016). Success rate and risk factors of failure of the induced membrane technique in children: a systematic review. Injury.

[16] Hsu CA, Chen SH, Chan SY, Yu YH (2020). The Induced membrane technique for the management of Segmental Tibial defect or Nonunion: a systematic review and Meta-analysis. Biomed Res Int.

[17] dos Reis FB, Faloppa F, Fernandes HJ, Albertoni WM, Stahel PF (2009). Outcome of diaphyseal forearm fracture-nonunions treated by autologous bone grafting and compression plating. Ann Surg Innov Res.

[18] Ring D, Allende C, Jafarnia K, Allende BT, Jupiter JB (2004). Ununited diaphyseal forearm fractures with segmental defects: plate fixation and autogenous cancellous bone-grafting. J Bone Joint Surg Am.

[19] Pesciallo CA, Garabano G, Dainotto T, Ernst G (2021). Masquelet technique in post-traumatic infected femoral and tibial segmental bone defects. Union and reoperation rates with high proportions (up to 64%) of allograft in the second stage. Injury.

[20] Sun H, Godbout C, Hali K, Momic J, Schemitsch EH, Nauth A (2022). The induced membrane technique in animal models: a systematic review. OTA Int.

[21] Arens D, Wilke M, Calabro L, Hackl S, Zeiter S, Zderic I et al. A rabbit humerus model of plating and nailing osteosynthesis with and without Staphylococcus aureus osteomyelitis. Eur Cell Mater. 2015;30:148 – 61; discussion 61 – 2. 10.22203/ecm.v030a11 26388617

[22] Metsemakers WJ, Schmid T, Zeiter S, Ernst M, Keller I, Cosmelli N (2016). Titanium and steel fracture fixation plates with different surface topographies: influence on Infection rate in a rabbit fracture model. Injury.

[23] Moriarty TF, Campoccia D, Nees SK, Boure LP, Richards RG (2010). In vivo evaluation of the effect of intramedullary nail microtopography on the development of local Infection in rabbits. Int J Artif Organs.

[24] Whelan DB, Bhandari M, Stephen D, Kreder H, McKee MD, Zdero R, Schemitsch EH (2010). Development of the radiographic union score for tibial fractures for the assessment of tibial fracture healing after intramedullary fixation. J Trauma.

[25] Leow JM, Clement ND, Simpson A (2020). Application of the Radiographic Union Scale for tibial fractures (RUST): Assessment of healing rate and time of tibial fractures managed with intramedullary nailing. Orthop Traumatol Surg Res.

[26] Giannoudis PV, Faour O, Goff T, Kanakaris N, Dimitriou R (2011). Masquelet technique for the treatment of bone defects: tips-tricks and future directions. Injury.

[27] Wu H, Shen J, Yu X, Fu J, Yu S, Sun D, Xie Z (2017). Two stage management of Cierny-Mader type IV chronic osteomyelitis of the long bones. Injury.

[28] Morris R, Hossain M, Evans A, Pallister I (2017). Induced membrane technique for treating tibial defects gives mixed results. Bone Joint J.

[29] Grun W, Hansen EJJ, Andreassen GS, Clarke-Jenssen J, Madsen JE. Functional outcomes and health-related quality of life after reconstruction of segmental bone loss in femur and tibia using the induced membrane technique. Arch Orthop Trauma Surg. 2022. 10.1007/s00402-022-04714-9 PMC10374690 36460763

[30] Croes M, Kruyt MC, Boot W, Pouran B, Braham MV, Pakpahan SA (2019). The role of bacterial stimuli in inflammation-driven bone formation. Eur Cell Mater.

[31] Croes M, Boot W, Kruyt MC, Weinans H, Pouran B, van der Helm YJM (2017). Inflammation-Induced Osteogenesis in a rabbit tibia model. Tissue Eng Part C Methods.

[32] Croes M, van der Wal BCH, Vogely HC (2019). Impact of bacterial Infections on Osteogenesis: evidence from in vivo studies. J Orthop Res.

[33] Lu W, Zhao R, Fan X, Wang H, Zeng M. Time-varying characteristics of the induced membrane and its effects on bone defect repair. Injury. 2022. 10.1016/j.injury.2022.12.026 36581479

[34] Toth Z, Roi M, Evans E, Watson JT, Nicolaou D, McBride-Gagyi S (2019). Masquelet technique: effects of Spacer Material and Micro-topography on factor expression and bone regeneration. Ann Biomed Eng.

[35] Gruber HE, Gettys FK, Montijo HE, Starman JS, Bayoumi E, Nelson KJ (2013). Genomewide molecular and biologic characterization of biomembrane formation adjacent to a methacrylate spacer in the rat femoral segmental defect model. J Orthop Trauma.

[36] Gruber HE, Riley FE, Hoelscher GL, Bayoumi EM, Ingram JA, Ramp WK (2012). Osteogenic and chondrogenic potential of biomembrane cells from the PMMA-segmental defect rat model. J Orthop Res.

[37] Wu H, Tan J, Sun D, Wang X, Shen J, Wang S (2023). Discovery of multipotent progenitor cells from human induced membrane: Equivalent to periosteum-derived stem cells in bone regeneration. J Orthop Translat.

[38] Christou C, Oliver RA, Yu Y, Walsh WR (2014). The Masquelet technique for membrane induction and the healing of ovine critical sized segmental defects. PLoS ONE.

[39] Fenelon M, Etchebarne M, Siadous R, Gremare A, Durand M, Sentilhes L (2021). Comparison of amniotic membrane versus the induced membrane for bone regeneration in long bone segmental defects using calcium phosphate cement loaded with BMP-2. Mater Sci Eng C Mater Biol Appl.

[40] Meng ZL, Wu ZQ, Shen BX, Li HB, Bian YY, Zeng L (2019). Reconstruction of large segmental bone defects in rabbit using the Masquelet technique with alpha-calcium sulfate hemihydrate. J Orthop Surg Res.

[41] Viateau V, Guillemin G, Calando Y, Logeart D, Oudina K, Sedel L (2006). Induction of a barrier membrane to facilitate reconstruction of massive segmental diaphyseal bone defects: an ovine model. Vet Surg.

[42] Liu H, Hu G, Shang P, Shen Y, Nie P, Peng L, Xu H (2013). Histological characteristics of induced membranes in subcutaneous, intramuscular sites and bone defect. Orthop Traumatol Surg Res.

[43] Gouron R, Petit L, Boudot C, Six I, Brazier M, Kamel S, Mentaverri R (2017). Osteoclasts and their precursors are present in the induced-membrane during bone reconstruction using the Masquelet technique. J Tissue Eng Regen Med.

[44] Arican G, Ozmeric A, Firat A, Kaymaz F, Ocak M, Celik HH, Alemdaroglu KB (2022). Micro-ct findings of concentrated growth factors (cgf) on bone healing in masquelet’s technique-an experimental study in rabbits. Arch Orthop Trauma Surg.

[45] Cho JW, Kim BS, Yeo DH, Lim EJ, Sakong S, Lim J (2021). 3D-printed, bioactive ceramic scaffold with rhBMP-2 in treating critical femoral bone defects in rabbits using the induced membrane technique. J Orthop Res.

[46] Roukoz S, El Khoury G, Saghbini E, Saliba I, Khazzaka A, Rizkallah M (2020). Does the induced membrane have antibacterial properties? An experimental rat model of a chronic infected nonunion. Int Orthop.

[47] Shah SR, Smith BT, Tatara AM, Molina ER, Lee EJ, Piepergerdes TC (2017). Effects of local antibiotic delivery from Porous Space maintainers on Infection clearance and induction of an osteogenic membrane in an infected bone defect. Tissue Eng Part A.

[48] Draeger RW, Dahners LE (2006). Traumatic wound debridement: a comparison of irrigation methods. J Orthop Trauma.

[49] Nair R, Schweizer ML, Singh N (2017). Septic arthritis and prosthetic joint Infections in older adults. Infect Dis Clin North Am.

