# Supplementary material for: Comparative bone healing with induced membrane technique (IMT) versus empty defects in septic and aseptic conditions in a novel rabbit humerus model
Source: BMC Musculoskelet Disord. 2023 Nov 14;24:886. doi: 10.1186/s12891-023-07031-3 (PMC10644571; doi:10.1186/s12891-023-07031-3)
Supplement: Supplementary file 1 — Supplementary Material 1 [file 12891_2023_7031_MOESM1_ESM.docx]

**Supplemental Figures**


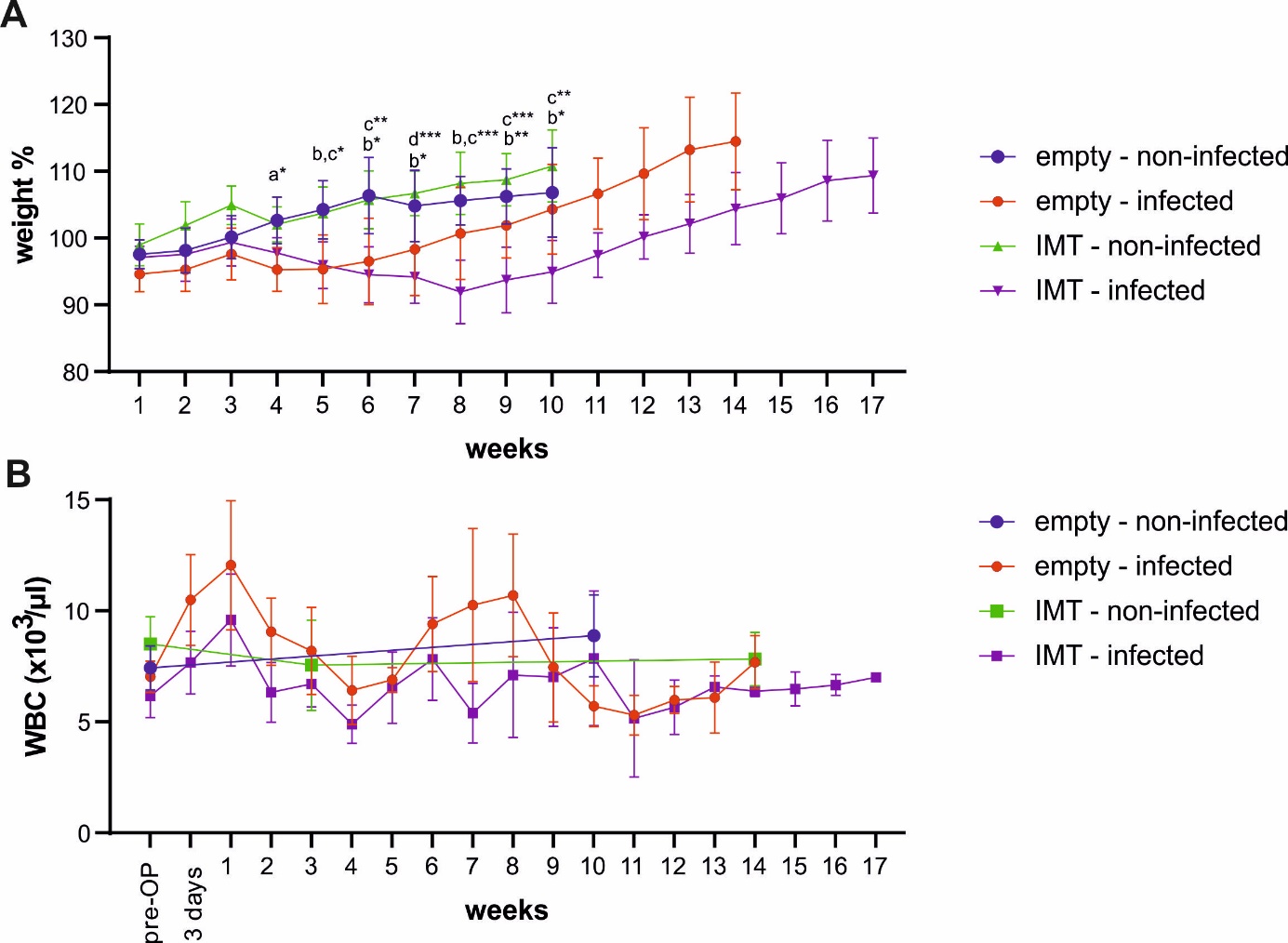


**Suppl. Fig. 1.** Animal welfare data. **A.** Body weight percentage of the animals during the study compared to the pre-study weight. Statistical analyses were performed using 2-way ANOVA with Šídák's multiple comparisons test, a=empty - non-infected *vs* empty infected, b= empty - non-infected *vs* IMT – infected, c= IMT – non-infected *vs* IMT – infected; **B.** White blood cell (WBC) count of the 4 groups pre-OP and 3 days after initial surgery and then every week. Statistical analyses were performed using 2-way ANOVA with Šídák's multiple comparisons test. No significant difference observed.


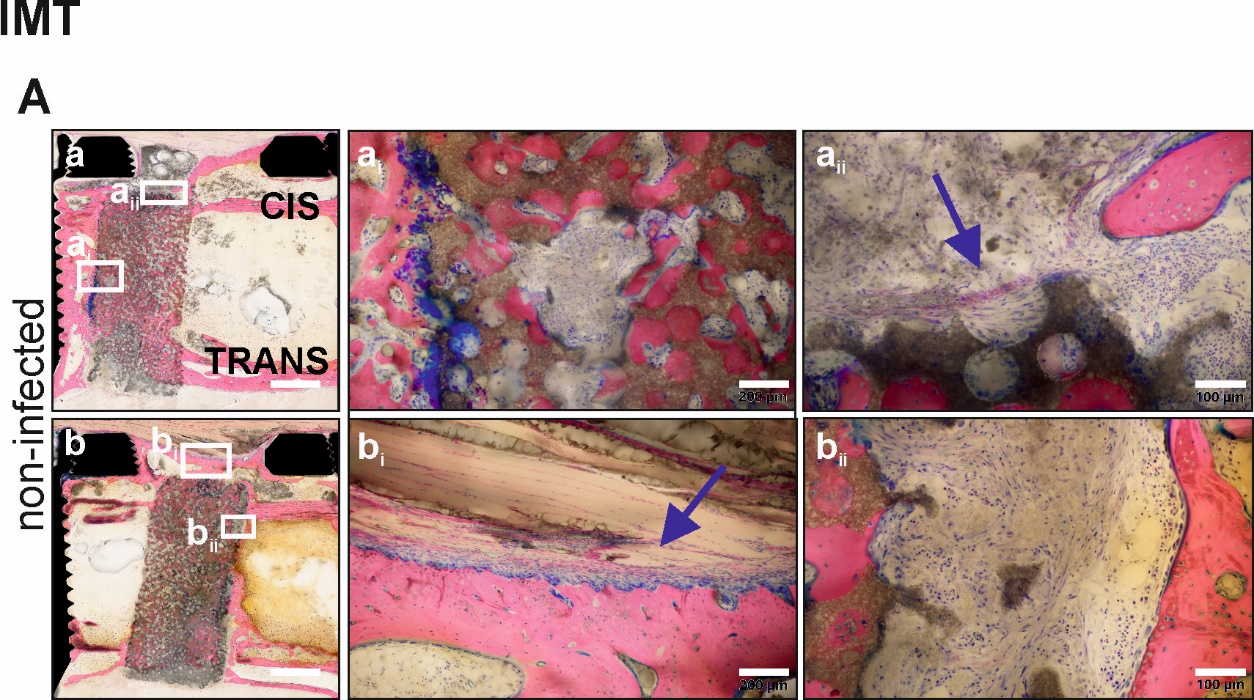


**Suppl. Fig. 2 A.** Microphotographs of the histopathological changes near the bone graft substitute in the IMT defect group. **A.** Two representative overview images of non-infected animals (*n*=6). b_ii_,: Note the presence of multinucleated giant cells with grey cytoplasmic material (interpreted as PMMA) as a sign of chronic granulomatous inflammation. Scale bar a and b 2 mm, a_i_, b_i_, 200 µm, a_ii_, b_ii_, 100 µm.


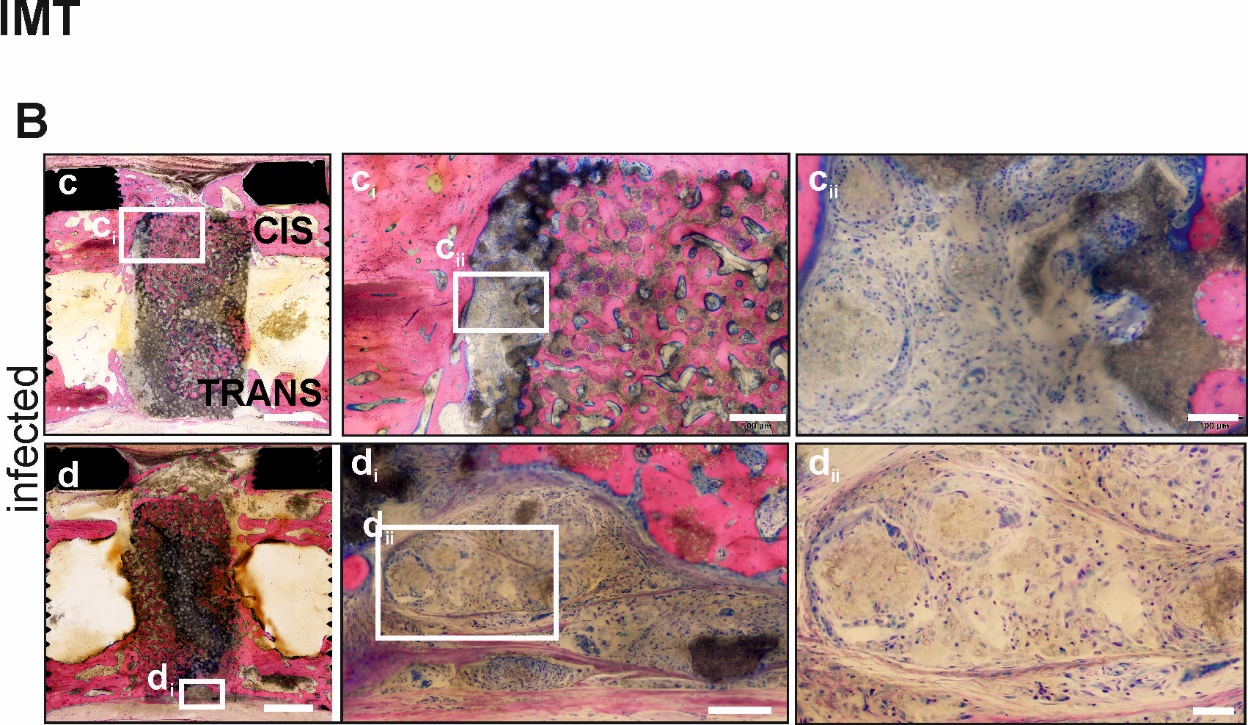


**Suppl. Fig. 2 B.** Microphotographs of the histopathological changes near the bone graft substitute in the IMT defect group **B.** Two representative overview images of infected animals (*n*=6). c_ii_ and d_ii_: note the presence of multinucleated giant cells with grey cytoplasmic material (interpreted as PMMA) as a sign of chronic granulomatous inflammation. Scale bar c and d 2 mm, c_i_ 500 µm, d_i_ 200 µm, c_ii_ 100 µm and d_ii_ 50 µm.


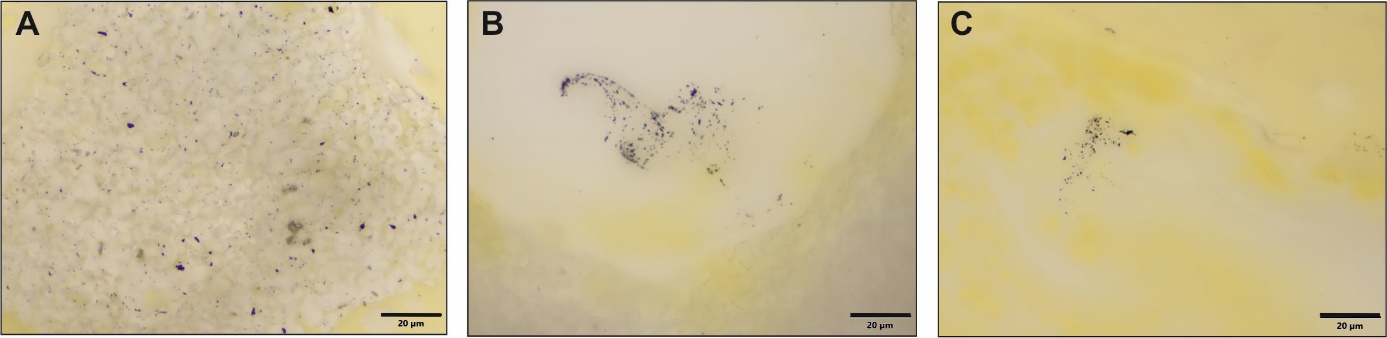


**Suppl. Fig. 3.** Representative microscopic images stained for BB of one animal from the infected IMT group. Note the presence of dark blue to black-stained, Gram-positive coccoid bacteria in the bone matrix. Objective 100x, scale bar 20 µm.
